# Lightweight Neural Networks-Based Safety Evaluation for Smart Construction Devices

**DOI:** 10.1155/2022/3192552

**Published:** 2022-06-15

**Authors:** Guimei Wang, Jianliang Zhou

**Affiliations:** ^1^School of Mechanics and Civil Engineering, China University of Mining and Technology, Xuzhou 221116, Jiangsu, China; ^2^Ascity Design Stock Co., LTD, Hangzhou 310015, Zhejiang, China

## Abstract

Based on the theory of lightweight neural networks, this paper presents a safety evaluation model for smart construction devices. The model index system includes the internal logical relationship between the input and output indexes, and the input indexes are specifically refined. According to the safety evaluation results, the article observes what type of accidents will occur at the construction site. According to the detailed and specific output index system, the six input factor layer indicators correspond to the indicators of several multiple network index layers, respectively. In the simulation process, MATLAB software was used to write the multiple neural network model program for the safety evaluation of the construction site, and the appropriate multiple network structure and related parameters were selected. The experimental results show that the multiple neural networks are trained by collecting 10 expert evaluation samples, and the trained multiple neural networks are applied to real construction cases. Comparing the two sets of data, it can be seen that the gap is relatively small, and the sample training is better. The multiple neural networks have relatively good evaluation performance. The method has a fast calculation speed and effectively improves the efficiency and practical value of safety evaluation.

## 1. Introduction

Judging from the historical process of social development in various countries in the world, the construction industry has always been an important pillar industry that affects the national economy and people's livelihood. The development and improvement of the construction industry not only present opportunities of the times, but also must face severe challenges [1–3]. Among them, due to the industry characteristics of the construction industry, there are many unsafe factors on the construction site, and the possibility of accidents is high. A little carelessness will cause heavy losses to the national economy and people's life and property safety. Therefore, the safety management of construction sites is a long-standing issue that needs to be faced for a long time and constantly improved. In recent years, with the healthy development of the regional construction industry and the steady improvement of the level of safety management, the number of construction safety accidents has been significantly reduced, but there is still a significant gap compared with the western developed countries [4–6]. After research, this paper decided to use the combination of expert scoring and AHP to obtain more objective, accurate, and real sample data, which is very important for the follow-up research work [7–9].

The safety evaluation of high-rise building construction site is not a linear model, but nonlinear. Multiple neural networks have many incomparable advantages over the traditional evaluation methods. These advantages will be an innovation in the construction safety evaluation of high-rise buildings. Its superiority lies in overcoming the defects of the old safety evaluation method, bringing a new evaluation data processing mode, and bringing accuracy and speed into the results of high-rise building construction safety evaluation [10, 11]. Secondly, through the research on the relevant content of the safety evaluation theory, the shortcomings of the commonly used evaluation methods are analyzed, and the safety evaluation research of high-rise building construction based on fuzzy mathematics is established. Based on expert opinions, a safety evaluation index system for high-rise building construction was constructed; finally, a safety evaluation model based on fuzzy mathematics was constructed, and the evaluation model was verified by using multiple neural networks [12–15].

This paper starts with safety management, applies the relevant theories and methods of artificial neural network to quantitative safety evaluation management work, and establishes a construction site dynamic safety evaluation management system, in order to grasp the safety status and evaluation of the construction site in real time during the construction process. This paper starts from the attributes and safety requirements of the construction site to identify the hazard sources, uses the fishbone diagram analysis method and the LEC evaluation method to evaluate the major hazard sources of the construction site, and proposes a feasible control scheme. The reliability of personnel in the man-machine system of the construction site is analyzed by the THEBP method, and a method to reduce the occurrence of human errors is proposed. Through the comparison of various evaluation methods such as accident tree analysis, event tree analysis, fuzzy mathematics comprehensive evaluation method, and multiple neural network method, it is found that the multiple neural network method is a simpler and more scientific method. First, this paper takes the relevant influencing factors of construction site safety as a factor set, the research status of building construction safety management at home and abroad is reviewed, the relevant theories of safety management are introduced, the commonly used safety evaluation methods are summarized and analyzed, their respective advantages and disadvantages are compared, and the dynamic safety of construction site is proposed.

## 2. Related Work

System safety engineering has been continuously promoted and developed in many fields, and has become a new modern system safety engineering method and theoretical system. It has a very important position in today's safety science. Safety evaluation is accepted by more and more enterprises; they gradually have their own evaluation methods, make predictions in advance, evaluate and analyze the safety and reliability of the system, and avoid losses as much as possible. This method is widely used at home and abroad. This method relies on human experience and judgment to identify potential safety hazards at the construction site, and reflects the survey results in a table, then review and check against the items in the table. The safety checklist method is static, intuitive, simple, and easy to use [16–18].

The disadvantage of AHP is that it cannot analyze complex systems, because some layers can affect other layers, and in turn other layers can affect it [19]. This kind of problem is more complicated, and the best way is to build a network model to deal with it. Even so, AHP is still a very good method, and its basic idea can play a very important role in establishing the evaluation criteria system. The hazard-causing structure and performance relationship of the most basic unit elements can be obtained, so that the probability of accident occurrence can be deduced and calculated, and the degree of the dangerous disaster can be obtained through estimation. The first step in doing fuzzy evaluation is to establish a set of influencing factors, and then give weights to each factor. Each factor is evaluated by establishing an evaluation set, so that an evaluation matrix can be obtained. Finally, the evaluation matrix and the weights form a system evaluation matrix so that the total score is calculated and then referred to the security level [20]. When the fuzzy comprehensive evaluation method deals with the variable weight and fixed weight of the factor weight, this method has its own limitations, because the problem of membership function and membership degree must be handled and solved manually.

Regona et al. [21] used the AHP to analyze the influencing factors affecting the safety construction of construction enterprises, and made an evaluation based on this, and gave corresponding suggestions. Hammad et al. [22] proposed to introduce momentum factor into artificial neural network to improve safety evaluation. Through the analysis of factors with the help of momentum factor, the safety evaluation is made more perfect and reasonable on the basis of artificial neural network. Mamun et al. [23] used a comprehensive evaluation method to evaluate the safety of building construction. Through the combination of multiple evaluation methods, the insufficiency of using a single method is avoided, the construction is objectively evaluated, and detailed countermeasures are given. Kim et al. [24] pointed out that the idea of PDCA was applied to the process of safety management, and he conducted an in-depth research on the safety management of construction enterprises, and realized effective safety control with the help of the idea of PDCA. The researchers introduced the risk analysis theory into the safety control of high-rise building construction, and by using the risk identification method to analyze the main factors affecting the construction of high-rise buildings, the occurrence of risks can be effectively controlled [10, 25, 26].

## 3. Evaluation of Multiple Neural Networks in Smart Construction Sites

### 3.1. Smart Construction Site Data Collection

For the smart construction site data set, it is generally set as a total representing the research objects. It is usually denoted by *U*. The relationship between elements and sets is divided into inclusion and noninclusion in the collection of smart site data sets, which are generally represented by feature functions. In general, membership functions can be used to describe fuzzy subsets. If it becomes an ordinary subset, then U must have a corresponding value range, and then its membership function is transformed into an ordinary characteristic function.

(1) $$\begin{array}{c} F_{i}\left(x,i\right)=\left\{f\left(i\right)-i,f\left(i-i\right)-i-1,f\left(i-2\right)-i-2,\ldots ,f\left(1\right)-1\right\}. \end{array}$$

When training multiple neural networks, a set of initial values of each connection is given, and the calculation is performed according to a certain method, and the final network weights are determined. Therefore, using the trained neural network for construction safety evaluation, the results are generally different. If the difference between the evaluation results is too large with the difference of the initial weights, the network is said to be unstable at this time. If the difference between the results in practical application is relatively small, the network in Table 1 is relatively stable.

However, the above results are not ideal. If the absolute error can be controlled within ±0.5 points, the effect is the best. However, if you continue to increase the number of neurons, you may not be able to obtain a more ideal learning effect. Taking the 14 × 1 type network as an attempt, after 5000 steps are counted, the final error of the system stays at 0.000334, which is not as good as the 8 × 1 type for the web. The simulation score of the first group of samples is 92.2030 points, and the fifth group of samples has a simulation score of 92.1336 points, and the error increases significantly.

(2) $$\begin{array}{c} u\left(i,j\right)=\sum_{i,j=1}\frac{f\left(i\right)}{f\left(j\right)}-\frac{u\left(i\right)}{u\left(j\right)}-\sum_{i,j=1}\frac{f\left(i\right)-1}{f\left(j\right)}-\frac{u\left(i\right)-1}{u\left(j\right)}. \end{array}$$

If the value of *i* is too large, the language is difficult to describe and it is difficult to judge the rank attribution; if the value of *m* is too small, it does not meet the quality requirements of fuzzy comprehensive evaluation. Usually, mode *i* takes an odd number in many cases, because it can have an intermediate level, which is convenient for judging the level of the object to be evaluated. The interaction strength between the current neuron and the *f*th neuron is shape (that is, the connection weight of the current neuron with respect to the *i*th neuron). Then, the input of neuron information this time can be expressed as *s*.

### 3.2. Multiple Neural Network Layers

Multiple neural network connections are called excitation functions or action functions. This function is a specific function that determines the output *i* = 1, which is usually nonlinear. The commonly used nonlinear excitation functions are threshold type and piecewise linear type.

(3) $$\begin{array}{c} t\left(i,j\right)=\sum_{i,j=1}\frac{f^{t-i}\left(i\right)}{f^{t-j}\left(j\right)}\sum_{i,j=1}\frac{u^{t-i}\left(i\right)}{u^{t-j}\left(j\right)}-\frac{u\left(i\right)}{u\left(j\right)}. \end{array}$$

The learning method with a tutor is that when the network inputs a sample, the tutor provides an expected output *d* to the network system, and adjusts the parameters of the network based on the error between the actual output value of the network and the expected output value *d*, that is, using that the reward and punishment scheme modifies the weight matrix and threshold matrix of the network, and this learning method requires a priori training subset.

**Figure 1:**
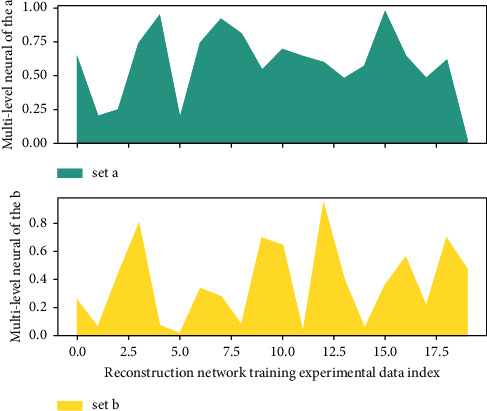
Multi-level neural network training.

When there is information input into the neural network of Figure 1, the information is first transmitted from the input layer node to the hidden layer node of the first layer, and after the function of the feature function, it is transmitted to the next hidden layer, and so on layer by layer. Finally, it is passed to the output layer for output. The excitation function of each layer is required to be differentiable, and a sigmoid function is generally used. The most basic multiple neural network is a feedforward network that includes three layers of nodes: input layer, hidden layer, and output layer.

(4) $$\begin{array}{c} g\left[t\left(i\right)-t\left(j\right)\right]-g\left[\frac{i\left(t\right)}{j\left(t\right)}-\frac{j\left(t\right)}{i\left(t\right)}\right]-1=0. \end{array}$$

When the information input is processed, the neural network itself can self-learn and adjust itself during the learning process to achieve the ultimate goal. The purpose of neural network adaptive self-learning is to verify the error between the actual output value of its output layer and the known output value, so as to minimize it. During the adaptive self-learning process, the error value gradually decreases until it reaches the allowable value. In the calculation process, the state of each layer of neurons only affects the state of the next layer of neurons, if the result obtained in the output layer is not the expected output, then it will be back-propagated. Backpropagation returns the error signal along the original connection path, and appropriately modifies the connection weights of neurons in each layer according to certain principles until the first hidden layer, and then starts forward propagation, using the input just now. The information is calculated by the forward network. If the output of the network meets the error requirement, the learning process ends.

### 3.3. Smart Site Network Parameter Settings

The smart construction site network parameter device is installed on the inner side of the top of the hanging cage, and the rotating arm 4 is fixed on the top of the hanging cage by the hinge fulcrum 5. One end of the pull rod 3 is hinged with the rotating arm 4, and the other end is hinged with the pin bar 2. The cage is in the running state at the position shown in the paper. When loading and unloading the goods after the hanging cage stops, move the handle 6 clockwise, and the rotating arm 4 drives the pull rod 3 to push the pin bar 2 sleeve, which is stuck on the web of the bracket, supports the hanging cage, and plays a role in preventing falling. In order to prevent the operator from making mistakes and forgetting to withdraw the pin, another touch switch 7 is provided.

(5) $$\begin{array}{c} f\left[\sum_{i,j=1}\frac{f\left(i\right)}{f\left(j\right)}-t\left(i,j\right)\right]-\sum_{i,j=1}\frac{f\left(i\right)-t\left(i,j\right)}{f\left(j\right)}=0. \end{array}$$

When the pin is outstretched, the power switch 7 is disconnected. Only when the pin is pulled back, the switch is reset, and the power is turned on, the construction site can run, so as to prevent the wire rope from being broken by misoperation or damage to the equipment parts.

**Figure 2:**
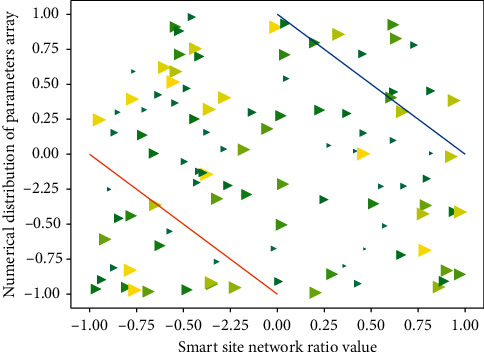
Network parameter distribution of smart construction site.

According to the order of the number (order) of the bottom events in the minimum cut set, under the condition that the occurrence probability of each bottom event is relatively small and there is little difference between each other, the minimum cut sets can be compared according to the following principles: the smaller the order of Figure 2 is, the more important the minimum cut set is; the bottom event that appears in the low-order minimum cut set is more important than the bottom event in the high-order minimum cut set; under the condition that the minimum cut set order is the same, the more times it occurs repeatedly in different minimum cut sets.

(6) $$\begin{array}{c} \sum_{i,j=1}\frac{f\left(i\right)-\exp\left(i,j\right)}{1-\exp\left(i,j\right)}-f\left(\frac{1+\exp\left(i,j\right)}{1-\exp\left(i,j\right)}\right)=0. \end{array}$$

The bottom event is more important. Among the mechanical equipment factors, the three indicators have the same weight, indicating that the loading and unloading of mechanical equipment, the reliability of vertical transportation equipment, and the repair and maintenance of mechanical equipment are all very important, and all affect the safety of high-rise building construction sites. Among the technical factors, the weight of the construction organization design is the highest, accounting for 0.5390, which tells the construction enterprise that the construction organization design must be done well, and cannot be simply pretended, because a good construction organization design can effectively avoid many high-level occurrence of construction safety accidents.

### 3.4. Multiple Neural Network Activation Functions

**Figure 3:**
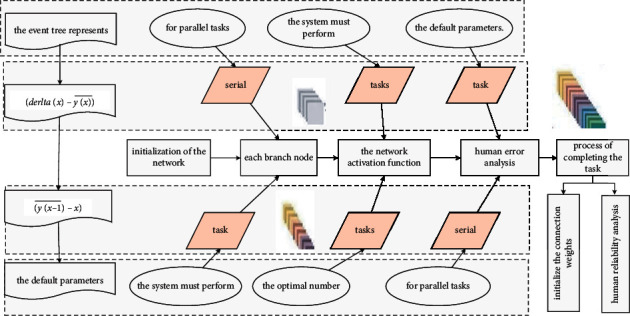
Multiple neural network activation function topology.

When using the network activation function for human error analysis, each branch node has two possibilities of failure or success. The systematic task analysis shows that: assuming that the human task includes subtask A, subtask B, and subtask C, the criterion for a person to successfully complete the task may be that the subtasks are connected in series or in parallel. For serial tasks, the system is required to successfully complete two subtasks A, B, and C at the same time; for parallel tasks, the system will succeed as long as one subtask is successfully completed. To build a human reliability analysis event tree, the task sequence should be decomposed according to the system requirements. Each bifurcation of the event tree represents the subtasks in Figure 3 that the system must perform in the process of completing the task.

Initialization is to initialize the connection weights and thresholds. When the new function establishes the network object, it automatically calls the initialization function to initialize the connection weights and thresholds of the network according to the default parameters. Using the function to customize the initialization of the network, and by selecting the parameters of the initialization function, the connection weights and thresholds of each layer can be initialized differently. After the multiple neural network is initialized, it can be trained. The range of finding the optimal number of intermediate nodes can be narrowed through empirical formulas. According to practical experience, the most suitable number of intermediate nodes has the strongest correlation with the number of input layer nodes.

(7) $$\begin{array}{c} \forall \left\{\tanh\left[a\left(i,j\right)-\tanh\left(a\right)=0\right)\right\},\\ \exists \left\{\frac{\tanh\left(a\right)}{\tanh\left(a\right)-1}⟶1\right\}. \end{array}$$

Therefore, the first method to reduce the number of intermediate nodes is: the method is to think that the optimal number of nodes in the middle layer is about 75% of the number of nodes in the input layer, and the input nodes in this paper are 5. According to this empirical formula, the number of nodes in the middle layer is 3, 4, and 5. The way is to try, and the workload is also relatively small.

## 4. Construction of a Comprehensive Evaluation Model for Construction Safety Based on Multiple Neural Networks in Smart Construction Sites

### 4.1. Smart Construction Site Safety Evaluation

Generally speaking, with the gradual increase of the number of hidden layer nodes in the network, the network performance can be significantly improved until a more suitable number of nodes are found and the optimal network structure is determined. If the number of nodes in the neural network continues to increase, until it has a particularly large number of neurons, it is still unable to obtain a satisfactory learning effect, we should consider adding a layer of hidden layer neurons, such as increasing the three-layer network to a four-layer network, the learning effect can be greatly improved, and the training time may be shorter than a three-layer network with too many nodes.

**Figure 4:**
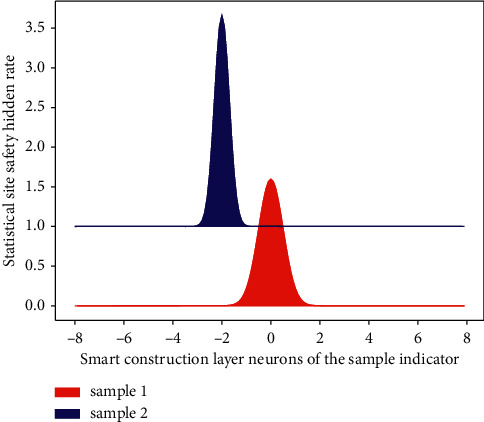
Evaluation of hidden layer neurons in smart construction site safety.

The output indicators of Figure 4 have been proposed, namely, falling from a height, hitting objects, collapsing, mechanical damage, lifting damage, and electric shock damage.

(8) $$\begin{array}{c} \langle \begin{array}{c} \frac{\tanh\left(a\right)}{\text{tan}h\left(a\right)-1}\\ \frac{u\left(a\right)}{u\left(a\right)-1} \end{array}\rangle ⟶\langle \begin{array}{c} \frac{f\left(a-1\right)}{f\left(a\right)}\\ \frac{1}{a} \end{array}\rangle . \end{array}$$

The 11 samples in this paper are all high-rise building projects that have been completed or are nearing completion, and the occurrence of these six major accidents can be judged according to the actual situation. The biggest difference between this method and the maximum membership degree method is that all the evaluation vectors are retained, and the distribution shows the state of each influencing factor. Decision-makers can comprehensively consider the impact of various factors on the evaluation object. However, it cannot effectively and accurately make judgments due to the existence of multiple factors, which is also the main disadvantage of this method.

(9) $$\begin{array}{c} \frac{\exp\left[av\left(i\right)\right]-\exp\left[-av\left(i\right)\right]}{\exp\left[av\left(i\right)\right]+\exp\left[-av\left(i\right)\right]}=\frac{\exp\left(av\right)}{\sum \exp\left[av\left(i\right)\right]v\left(i\right)}. \end{array}$$

Then, the error is analyzed by comparing the output result with the expected result, and if the error is within a predetermined range, the learning ends. Finally, use the reserved test sample to test the network, and the output result is the same as the expected result. The neural network using the data obtained from the fuzzy comprehensive evaluation as the sample meets the requirements, thus verifying the feasibility of the evaluation.

### 4.2. Exponential Dependence of Multiple Neural Networks

Multiple neural network index perceptron network structure with one hidden layer can accurately simulate any continuous function. For discontinuous signals such as square wave and sawtooth wave, two or more hidden layers are required to obtain better simulation results. For a three-layer multi-layer neural network, it is inappropriate to have too many or too few hidden layer nodes.

(10) $$\begin{array}{c} f\left(v\right)\times v\left(i\right)\text{d}v\text{d}i-v\left(i\right)\text{d}i=\frac{av\left(i\right)\text{d}i\times \;\exp\left(i\right)}{1+av\left(i\right)\text{d}i\times \;\exp\left(i+1\right)},\;\text{for}\left\{i=1,2,3,\ldots ,n\right\}. \end{array}$$

If there are too few hidden layer nodes, the ability of the neural network to obtain sample information is poor, and it cannot fully memorize and learn the various information and laws contained in the sample set; too many hidden layer nodes are not conducive to the neural network in the process of learning, and elimination of interference signals, and overfitting may occur. Too many hidden layer nodes will also increase the network training time and significantly reduce the network training speed.

(11) $$\begin{array}{c} \frac{\int av\left(i\right)\text{d}i-\exp\left(i\right)}{\int 1-av\left(i\right)\text{d}i+av\left(i\right)\text{d}i-\exp\left(i+1\right)}=\frac{\int av\left(i\right)\text{d}i}{\int 1-av\left(i\right)\text{d}i}. \end{array}$$

**Figure 5:**
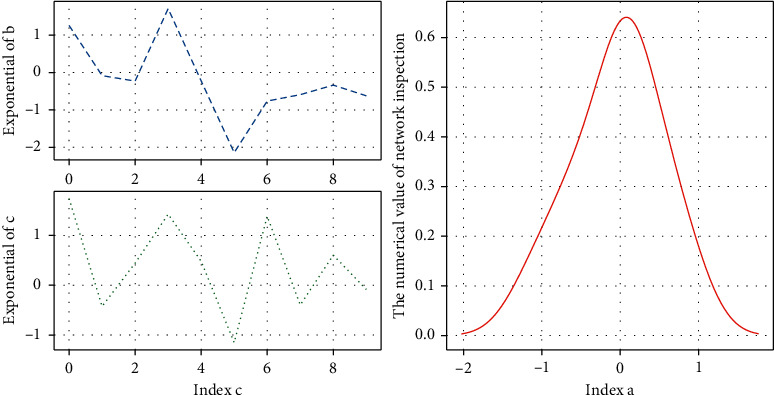
Distribution of exponentially dependent elements of multiple neural networks.

The input indicators of this paper are people, materials, mechanical equipment, technology, environment, and management factors. These 6 factors are selected as input vectors because these 6 factors constitute a complete system, complement each other, and can best describe the construction of high-rise buildings situation on-site. These six factors contain different indicators, and the weight of the indicator layer is determined. This article invited five experts from a large construction enterprise in the region. The names of these five experts are not described here for confidentiality reasons. The index score of each factor is multiplied by its respective weight, that is, the score of each factor is calculated by weighting, and this score is the input parameter of Figure 5.

Each artificial neuron that constitutes the neural network is a small “calculator” for processing problems. Each neuron operates independently according to its own input signal, then outputs the result, and then accepts new input signals, and continues to loop until the network runs. The powerful parallel computing capability greatly speeds up the processing of complex problems. The highly parallel structure allows multiple complex information to be widely distributed in each connection weight of each neuron. When the information in memory needs to be used, the neural network is able to “associate” when stimulated by input information. Therefore, the information storage and processing of artificial neural network are parallel in time and distributed in space.

### 4.3. Comprehensive Analysis of Construction Safety

The training process of the network is the process of finding the mathematical relationship between the various data in the construction safety samples, and the final network state is completely determined by the provided samples. A typical sample can enable the network to quickly obtain the mathematical laws behind it, but if the sample composition is more complex, it may allow the neural network to absorb too many sample details in the process of learning, but interfere with the essential laws that the samples follow. The learning of the main rules may also produce overfitting. It can be seen that the weight value of the mechanical factor index in the first level is 0.4478, and the membership degree is the largest, so it can be seen that its safety level is in the first level of safety, indicating that the mechanical factor does not affect the safe construction of high-rise buildings.

(12) $$\begin{array}{c} y\left(i,j\right)=\left\{y_{i}^{j}\left(x\right)-x,y_{i-1}^{j-1}\left(x\right)-x,y_{i-2}^{j-2}\left(x\right)-x,y_{i-3}^{j-3}\left(x\right)-x,\ldots ,y_{i-n}^{j-n}\left(x\right)-x\right\}. \end{array}$$

**Figure 6:**
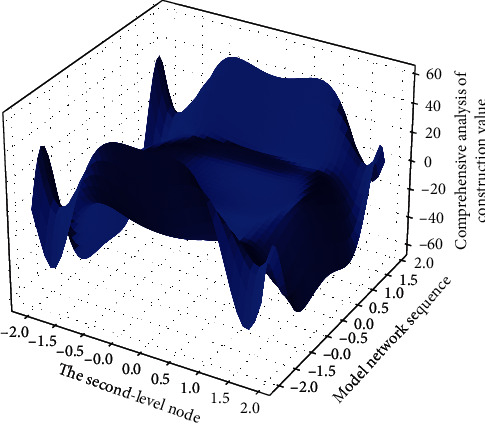
Comprehensive analysis of the second level of construction safety.

The weight value of the management factor index in the second level is 0.5526, and the degree of membership is the largest. Therefore, it can be seen that its safety level is safer in the second level, indicating that the management factor has no impact on the safe construction of high-rise buildings, but it also needs to pay more attention to prevent that the safety construction is affected by the management factors. The weight value of the factor index in Figure 6 in the second level is 0.5526, and the degree of membership is the largest. Therefore, it can be seen that its safety level is safer in the second level, indicating that the management factor does not affect the safe construction of high-rise buildings, but it also needs to be improved to prevent the influence of management factors on safe construction.

The pile foundation construction unit began to withdraw from the site, and the main structure construction unit entered the site. During this period of time, there was a large flow of personnel and machinery, the site conditions were complex, there were many cross-operations, the sources of danger increased, the difficulty of identification increased, and the safety quality of new personnel was uneven, and it is not conducive to the safety management of the construction site. Therefore, the safety situation of the construction site has decreased significantly.

At the same time, the supervision department has also strengthened the safety supervision of the site, urging the construction unit to strengthen the safety management, and new construction units entering the site. The safety system of the project has been established and implemented, the safety education of new workers has been strengthened day by day, and the safety situation of the project construction site has also improved day by day.

## 5. Application and Analysis of a Comprehensive Evaluation Model for Construction Safety Based on Multiple Neural Networks in Smart Construction Sites

### 5.1. Multiple Neural Network Data Pooling

There are two methods for data pooling training of neural networks: one is the incremental method, that is, each time a learning sample is input, the connection weights and thresholds are updated according to the network error; the other is the batch method, that is, only all learning after the samples are all learned, the network connection weights and thresholds are updated. The function train uses a batch method to update the network connection weights and thresholds. It is necessary to set its parameters, such as learning step size and error target. At the same time, during the network training process, the change of network error with the number of learning times is also displayed graphically.

(13) a∗yijx−x+b^2∗yijx−x+c^3∗yijx−x3+⋯+n=1−fvx,ix.

**Figure 7:**
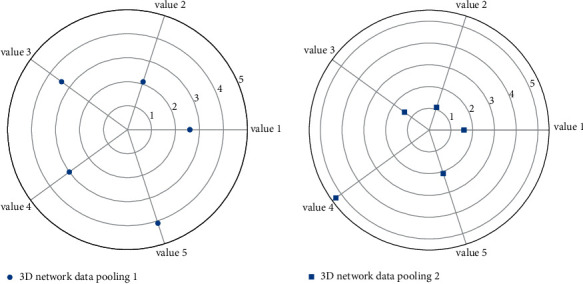
Data pooling distribution of multiple neural networks.

After the network structure is determined, it needs a certain training sample to be trained, and the sample should be widely representative. Field data collection is an important channel for effective sample selection. According to the characteristics of network learning, when selecting learning samples, a set of samples should be reserved for network detection to verify the feasibility of the network. After nearly 3 months of data collection and consultation with the person in charge of enterprise management and technical experts, a total of 9 sets of data were collected as the samples in Figure 7 to train the network, and 2 sets of samples were used for testing.

Through the study of the membership degree matrix, according to the principle of maximum membership degree, the membership interval of each evaluation index can be obtained, and which indicators are in a better interval and which indicators are in a poor interval, thereby affecting the safety of the proposed project and various indicators and factors for prevention and control, including the 0.1 scoring method and the 0–4 scoring method. It adopts certain scoring rules and adopts forced comparison scoring to evaluate the importance of the evaluation object. The difference in importance in the scoring method is only 1 point, and the grades cannot be opened. In order to make up for this deficiency, the classification is expanded to 4 levels.

(14) $$\begin{array}{c} \left\{\begin{array}{c} i\in \left[-1-m\left(x\right)\times x+n,0\right],\\ j\in \left[0,1-m\left(x\right)\times x-n\right]. \end{array}\right) \end{array}$$

When the 0–4 method is used, the evaluation functions are listed and compared one by one according to the importance of their respective functions. After the comparison, 4 points are awarded for very important ones, and 0 for very unimportant points, 3 points for more important, 1 point for less important, and 2 points for equally important or basically equally important.

### 5.2. Simulation Realization of Comprehensive Evaluation of Construction Safety

From the simulation results of comprehensive evaluation of construction safety, the first group of samples is [94; 95; 89; 89; 94; 88; 90; 92; 91; 94], and the evaluation result is 91 points. After simulation, the obtained evaluation score is 85.7621 points, with an error of 5.76%; for the fifth group of samples, the input vector value is [94; 89; 91; 86; 96; 92; 90; 94; 85; 90], the evaluation result is 90 points, and for the simulation based on the neural network after training, the evaluation score obtained is 85.4023 points, the error is 5.11%, and the error of the two groups of data is more than 5% for the full score. For a 100-point construction site safety evaluation management system, this error is unacceptable. We consider increasing the number of hidden layer nodes in Table 2, and use an 8 × 1 neural network for training.

Among them, TF represents the transfer function of the *i*th layer, which is an important combined part of the network model, which affects the running process of the model. Since it must have gradients during operation, these functions need to have derivative functions. The number of nodes in the middle layer increases one by one from a smaller number of nodes in the middle layer, or decreases from the number of nodes in the middle layer from a larger one, and so on, one by one, until a suitable number of nodes in the middle layer appear.

(15) $$\begin{array}{c} \frac{o\left(x,y\right)}{y\left(x\right)}=\frac{1}{1-x}\frac{\left(\text{derlt}a\left(x\right)-\bar{y\left(x\right)}\right)\left(\bar{y\left(x-1\right)}-x\right)}{y\left(x-1\right)}. \end{array}$$

There are 6 input nodes in this article. According to experience, the number of nodes in the middle layer is 4, 5, and 6. At this time, if we try one by one, the workload is not large. Another method is the empirical formula *x*, where *x* represents the number of intermediate nodes, *y* represents the number of input nodes, *i* represents the number of output nodes, and the value of a is 2 to 6, we put *x* = 6, *y* = 6 into the formula, the value of *z* is 5.46–9.46, rounded to 6–9. Combining these two empirical methods, the number of nodes in the middle layer can be determined as 6, so the structure of the multiple neural network is 6–6.6.

### 5.3. Example Application and Analysis

**Figure 8:**
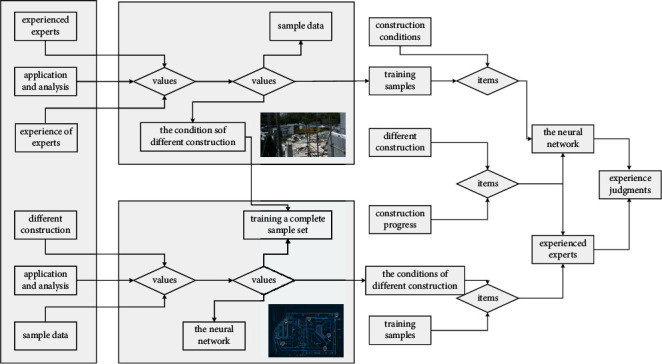
Comprehensive evaluation process of multinetwork construction security.

The training samples of the neural network are obtained by experienced experts in different construction sites, under the conditions of different construction progress and construction conditions. The sample data contains the experience judgments of the experts. Only by training a complete sample set can the judgment rules in the samples be effectively learned, and the rich experience of experts can be materialized into each connection weight of each neuron that constitutes the network. When the neural network is trained, and the network is used to evaluate the safety status of the site, the expert experience remembered by the network will play a role to guide the neural network to make appropriate judgments. For the neural network of Figure 8, the missing input items must have specific values to fill in the vacancies, otherwise the program cannot run.

The network structure adopts a 6 × 2 × 1 double hidden layer network structure, which has fast training speed, small error, stable network structure, and good simulation effect. The mean square error is only 4.99 × 10, and the simulation evaluation results of the training samples do not exceed 0.2 points. In view of the problem of how to deal with the incomplete samples, the existing theoretical methods of safety evaluation are generally unable to deal with incomplete samples, and must be supplemented with specific values. The missing items are replaced with 0 points, 70 points, and 100 points, respectively, and the input parameters are completed for evaluation and simulation. After analysis and comparison, it is believed that the use of 70 points to replace the missing items has the least impact on the final evaluation results, and can be considered partial safety, which meets the requirements of construction site safety evaluation management.

**Figure 9:**
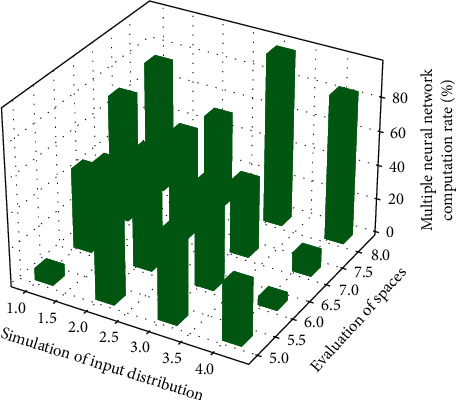
Simulation of multiple neural network activation input evaluation.

It can be clearly seen that the training effect of the network in Figure 9 has been significantly improved. Under the 8 × 1 neural network structure, MATLAB completed 5000 trainings in 37 seconds, and the final error was 0.000261, which was one-fifth of the final error of the 4 × 1 network. Then, examining the training results of the samples in this network: according to the neural network after training, the first group of samples scored 91 points, the simulation evaluation was 90.2877 points, the error was 0.78%, and the fourth group of samples scored 90 points. The simulation evaluation is 90.4423 points, the error is 0.749%, the error of the two groups of data is reduced to less than 1%, the training effect is good, and the absolute error is controlled within ±1%, which is basically acceptable in engineering practice.

## 6. Conclusion

This paper uses the powerful fault-tolerant ability of multiple neural networks to keep the network stable even when the input parameters have obvious deviations. Through the research on the relevant content of the safety evaluation theory, the shortcomings of the commonly used evaluation methods are analyzed, and the fuzzy mathematics is established for the theoretical basis of high-rise building construction safety evaluation research. Then, based on the principle of establishing evaluation indicators, combined with relevant regulations and expert opinions, a high-rise building construction safety evaluation index system is constructed; finally, a safety evaluation model based on fuzzy mathematics is constructed, and using that the evaluation model was verified by multiple neural networks. By using the established safety evaluation model to evaluate the construction enterprise instance, and using the evaluation results to analyze the aspects that the construction enterprise needs to improve. Considering filling the vacancies of the evaluation items with appropriate values, which will not significantly interfere with the evaluation results, let the neural network system identify its error attributes, and avoid repeated network construction, repeated training, and the emergence of problems such as the inability of the network after specific training to be generalized. Using the 11th sample of an office building to test the trained neural network, and continue to use MATLAB to test the neural network. In this paper, 0 points, 70 points of qualified points, and 100 points are considered to be substituted into the test sample parameters of the neural network, respectively. It may replace the missing evaluation items, observe the error between the evaluation result and the original comprehensive evaluation score, and finally decide which method to choose to deal with the missing items in the safety evaluation process.

## Figures and Tables

**Table 1:** Data collection of smart construction sites.

Data collection	Type network	System stay	Attempt rate/%	Number of neurons
Construction A	0.186	0.674	39.401	76
	0.322	0.380	46.608	10

Construction B	0.346	0.821	44.537	48
	0.638	0.527	28.231	42

Construction C	0.559	0.257	10.822	62
	0.295	0.202	22.793	9

**Table 2:** Multiple neural network simulations at the end of training.

Multiple neural network case	Test training simulations
Kinds = data.iloc[:, 0]	Of hidden layer nodes in *y*(*x* − 1)
Labels = data.iloc[:, 2:].columns	Increasing the number of hidden layer
Centers = pd.concat([data.iloc[:, 2:], data.iloc, axis = 1)	The first column of [*F*_*i*_(*x*) − *F*_*i*_(*y*)]
Plt.figure(figsize = (6, 4))	The second column is *y*_*i*_^*j*^(*x*) − *x*
Plt.contourf(*x*, *y*, *z*)	The last are those to *F*_*i*_(*x*)
Ax.plot(angles, centers[*i*], lw = 2, label = kinds[*i*])	Of each cluster $\bar{y\left(x-1\right)}$
Ax.fill(angles, centers[*i*])	Describe the center *av*(*i*)d*i*
*X* = np.arange(1, st.tot_det-1, st.step)	The number *x*, *y*, *z* = *x*_list, *y*_list, *z*_list
*Y* = np.arange(1, st.tot_det-1, st.step)	Management system derlta(*x*)
*X*, *Y* = np.meshgrid(*x*, *y*)	Consider increasing the number ∫*av*(*i*)d*i*
*Z* = np.mat(an)	This error is unacceptable

## Data Availability

The data used to support the findings of this study are available from the corresponding author upon request.
